# Supplementary material for: Fluvastatin Converts Human Macrophages into Foam Cells with Increased Inflammatory Response to Inactivated Mycobacterium tuberculosis H37Ra
Source: Cells. 2024 Mar 18;13(6):536. doi: 10.3390/cells13060536 (PMC10969755; doi:10.3390/cells13060536)
Supplement: Supplementary file 1 [file cells-13-00536-s001.zip › cells-2858743-supplementary.pdf]

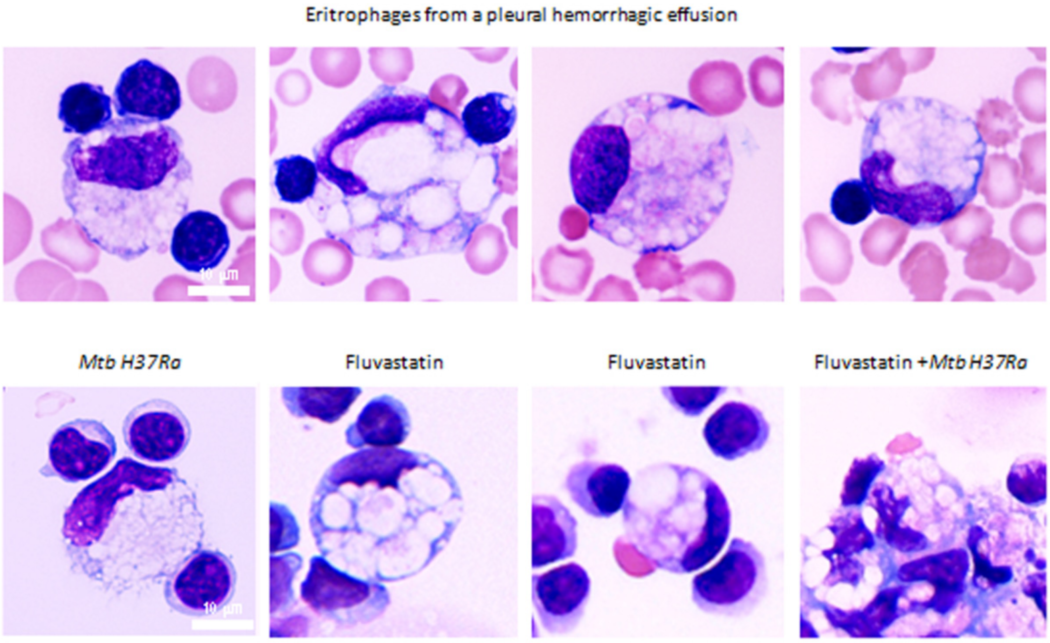

Figure S1. Analogies between erythrophages and in vitro-induced vacuolated macrophages. Microphotographs show the similarity between erythrophages from a sample of a pleural hemorrhagic effusion (upper row) and vacuolated macrophages induced in PBMC by the exposition to *Mtb H37Ra*, by fluvastatin treatment, or by the combination of both (low row). Note that all of them show a similar highly vacuolated cytoplasm, have displaced nuclei, and bind lymphocytes and monocytes. Moreover, like erytrophages, in vitro-induced vacuolated macrophages also capture erythrocytes.

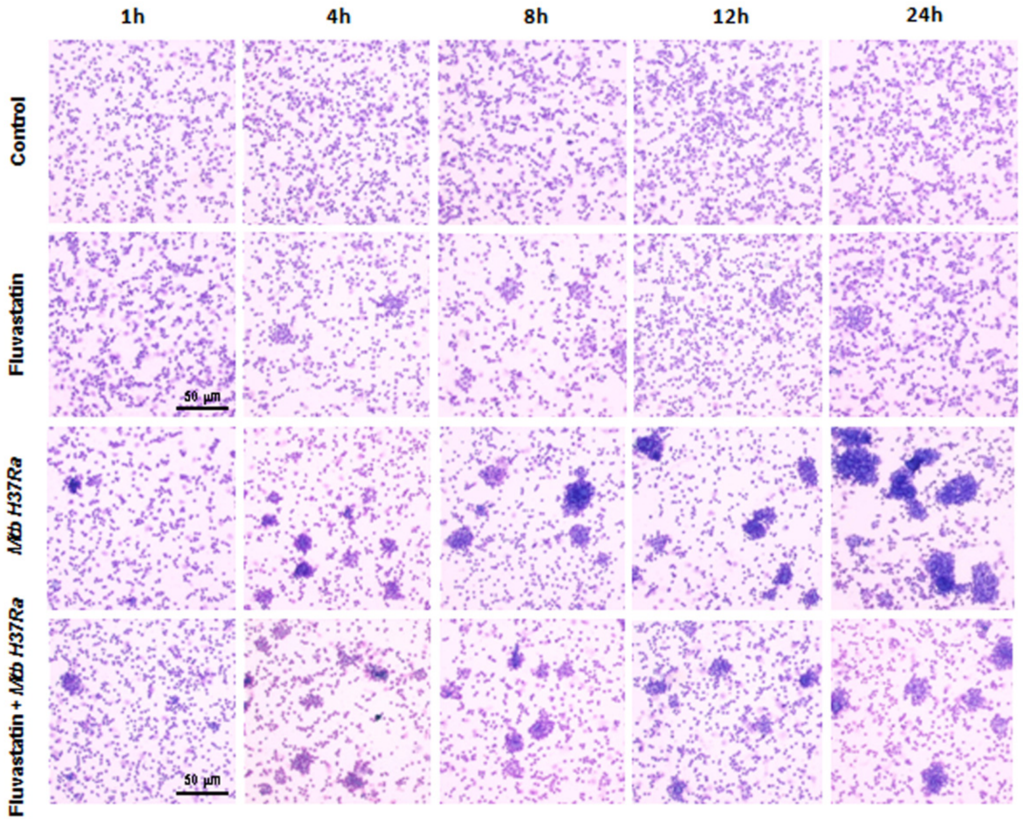

Figure S2. Evolution of cell cultures. Microphotographs show the evolution of the different cultures during the period of immune stimulation**.** Fluvastatin-treated PBMC show small cellular aggregates not observed in control conditions. After a few hours of exposition to *Mtb H37Ra*, PBMC show compact structures that grow over time and coalesce. Fluvastatin+ *Mtb H37Ra* treated cultures show numerous small and not compact aggregates.

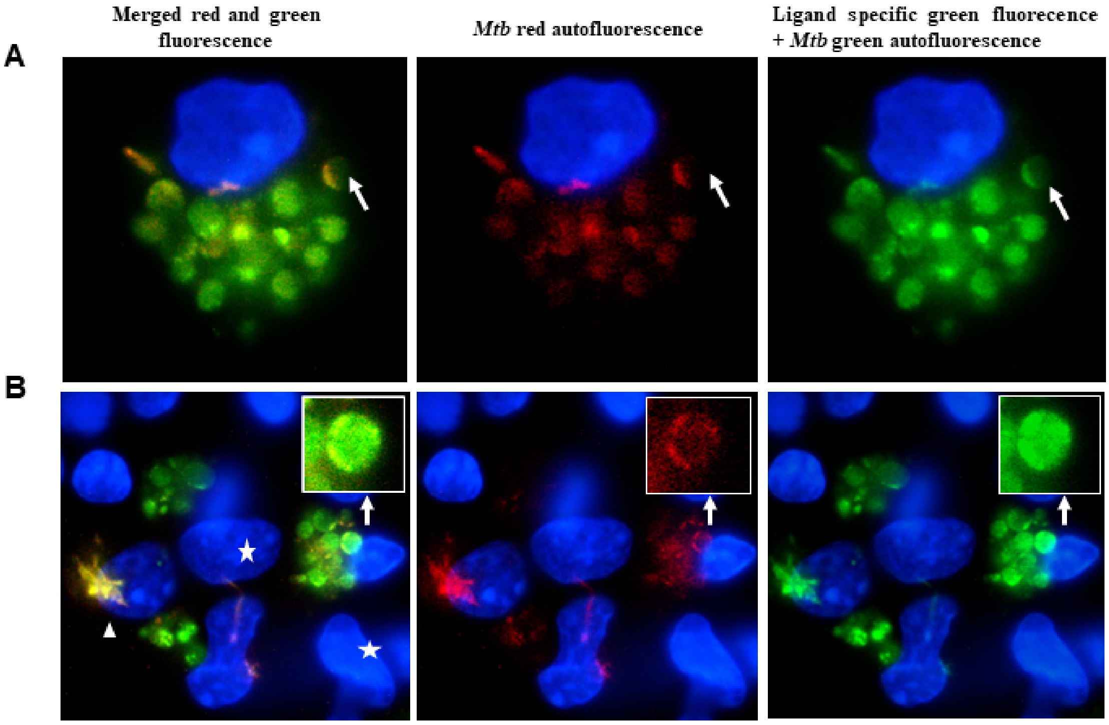

Figure S3. Caspase-1 activation in fluvastatin treated macrophages exposed to ***Mtb*.** A) The photomicrographs show the image presented in Figure 5D1 magnified and with the fluorescence of the green and red channels merged or not. B) This image shows the presence of several treated macrophages polarized by the bacteria to perform different functions in the same microenvironment. The image with merged fluorescence shows a macrophage that has captured large amounts of bacteria (in yellow), which apparently enters into its nucleus, without showing specific green fluorescence (white arrow head). Other macrophages (white starts) do not contain autofluorescent bacteria or their remnants, nor do they show specific green fluorescence. The remaining macrophages show a variable number of organelles enclosing both numerous fragments of degraded bacteria (red dots) and specific green fluorescence. In A and B, white arrows show some organelles enclosing active caspase-1 that accumulate bacilli remnants forming arcs at their edges.
